# Endovascular Treatment of Chronic Total Occlusion in the Subclavian Artery: A Review of 23 Cases

**DOI:** 10.3389/fneur.2020.00264

**Published:** 2020-04-24

**Authors:** Guochen Niu, Ziguang Yan, Bihui Zhang, Min Yang

**Affiliations:** Interventional Radiology and Vascular Surgery Department, Peking University First Hospital, Beijing, China

**Keywords:** subclavian artery stenting, subclavian steal syndrome, subclavian artery stenosis, chronic total occlusion, endovascular treatment

## Abstract

**Objectives and Background:** To review technical details and long-term outcomes of endovascular treatment of chronic total occlusion (CTO) of the subclavian artery.

**Methods:** From January 2010 to May 2017, 23 patients (17 male; median age, 65 years) underwent endovascular treatment for CTO of the subclavian artery. All lesions had been diagnosed by duplex scanning or computed tomography angiography before treatment. Sixteen (70.0%) patients had symptoms of vertebrobasilar insufficiency, 6 (26.1%) patients had symptoms of arm ischemia, and 2 (8.7%) patients were asymptomatic. Duplex scanning revealed that 16 patients had grade 3 and 7 patients had grade 2 steal blood flow. After recanalization, lesions were treated by stenting. Patients were followed up at 1, 3, 6, and 12 months after endovascular treatment, and annually thereafter.

**Results:** The overall technical success rate was 91.3% (21/23). The successful recanalization rate of antegrade and retrograde approaches were 68.2% (15/22) and 75.0% (6/8), respectively. The rate of clinical symptom remission was 95.2% (20/21) after treatment. No perioperative death or permanent neurological deficits were observed. One patient had arterial dissection treated by covered stent. The estimate cumulative primary and secondary patency rates at 5 years were 74.6 and 78.8%, respectively.

**Conclusion:** Endovascular treatment is a feasible and safe treatment for CTO lesions of the subclavian artery.

## Introduction

Subclavian steal syndrome is a systemic entity that was first reported in 1961 (1). It might lead to ischemic neurological symptoms. Patients with subclavian steal syndrome might have symptoms of vertebrobasilar insufficiency, such as transient ischemic attacks, vertigo, dizziness, syncope, and stroke (2, 3). Percutaneous transluminal angioplasty and stenting are minimally invasive approaches to treat subclavian artery stenosis. Clinical outcomes similar to surgical methods can now be achieved by endovascular treatment with minimal risks under local anesthesia (4, 5). Chronic total occlusion (CTO) is commonly recognized as the challenging lesion subset to be treated by percutaneous interventions. CTO lesions are more difficult to break through than non-occlusive stenosis, which decreases the technical success rate of the procedure. There are very few reports regarding endovascular treatment for CTO lesions of the subclavian artery. In this study, we reported 23 cases treated in our center to evaluate the safety and effectiveness of endovascular treatment for patients with CTO in the subclavian artery.

## Methods

### Patient Data

This study was approved by the Institutional Review Board of Peking University First Hospital. From January 2010 to May 2017, 23 patients were diagnosed with CTO in the subclavian artery by angiography and received endovascular treatment in our department (17 males; median age, 65 years). Demographics and clinical characteristics of these patients are summarized in Table 1.

**Table 1:** Demographics and characteristics of enrolled patients.

**Features**	**Mean ± SD or *n* (%)**
Age (y)	65 ± 10.1
Male	17 (73.9)
Family history of atherosclerosis	11 (47.8)
Hypertension	16 (69.6)
Diabetes mellitus	6 (26.1)
Coronary heart disease	7 (30.4)
History of stroke^*^	9 (39.1)
Smoking	15 (65.2)

* *including lacunar infarction*.

### Symptoms and Signs

In our study, 16 (70.0%) patients had symptoms of vertebrobasilar insufficiency including vertigo, syncope, and so on, 6 (26.1%) patients had symptoms of arm ischemia including arm numbness, arm claudication, and 2 (8.7%) patients were asymptomatic (Table 2).

**Table 2:** Symptoms and signs of the patients.

**Symptoms/Signs**	***n* (%)**
Vertigo	14 (60.9)
Syncope	2 (8.7)
Arm numbness	3 (13.0)
Arm claudication	5 (21.7)
Absent radial pulse^*^	2 (8.7)
Weak radial pulse^**^	21 (91.3)

*, ** *occurred on the affected side*.

### Imaging Examinations

Before endovascular therapy, every patient underwent duplex ultrasound and brain magnetic resonance imaging (MRI) scanning. Subclavian steal phenomenon was categorized into three levels based on vertebral artery flow patterns: Grade 1 refers to antegrade flow with systolic notch; Grade 2 refers to bidirectional blood flow; and Grade 3 refers to continuous retrograde flow (6). Sixteen patients had grade 3 and 7 patients had grade 2 vertebral steal blood flow on the affected side. For preoperative evaluation, 18 patients received computed tomography angiography (CTA) and 3 patients had magnetic resonance angiography (MRA). All lesions of the patients in our study were proximal to the origin of the vertebral artery.

### Medication

All patients had taken 100 mg of aspirin daily and 75 mg of clopidogrel daily for at least 3 days before the procedure. Otherwise, a loading dose of 300 mg of aspirin plus 300 mg of clopidogrel were given 6 h prior to the procedure. After stenting, all patients were placed on 100 mg of aspirin daily for lifelong therapy and 75 mg of clopidogrel daily for the range 3 to 6 months.

### Endovascular Therapy

All procedures were performed under local anesthesia. During the procedure, an activated clotting time of 200–300 s was maintained by intravenous administration of heparin. For each patient, a four-vessel cerebral angiography was used to evaluate the severity and location of lesions and reveal the patency of the posterior communicating artery and other anatomy abnormalities. For recanalization, the right femoral artery was chosen as the primary approach, while the ipsilateral brachial artery was used as an alternative retrograde approach if attempting to pass through the lesion via the femoral artery failed. For any reason, if transfemoral access was not feasible, we attempted recanalization via the ipsilateral brachial artery directly. If a lesion was located in the proximal segment of the right subclavian artery, a distal emboli protection device was deployed in the right carotid artery before we tried to breakthrough the lesion. After breaking through occlusive lesions, angiography was performed to confirm that the catheter was in the true lumen. Occlusive lesions were routinely predilated in all patients. Balloon-expandable stents were favored in the right subclavian artery, because these types of stents can facilitate precise deployment to avoid any obstruction of the right common carotid artery while covering the lesion completely. Both Balloon-expandable stents and self-expandable stents could be implanted in the left subclavian artery. Post-dilation of the stent was performed if residual stenosis was >50%. At the end of the procedure, angiography was performed to document the improvement of vertebral artery and basilar artery blood flow.

### Follow-Up

A carotid duplex ultrasound scan was performed in all patients at 1 and 3 months after treatment. CTA was performed at the 6 month follow-up. Patients with no significant restenosis or recurrent symptoms were followed annually thereafter. For patients with recurrent symptoms or suspected restenosis detected by noninvasive imaging workup, angiography was recommended. Endovascular treatment was suggested for confirmed restenosis.

### Definition

**Figure 1:**
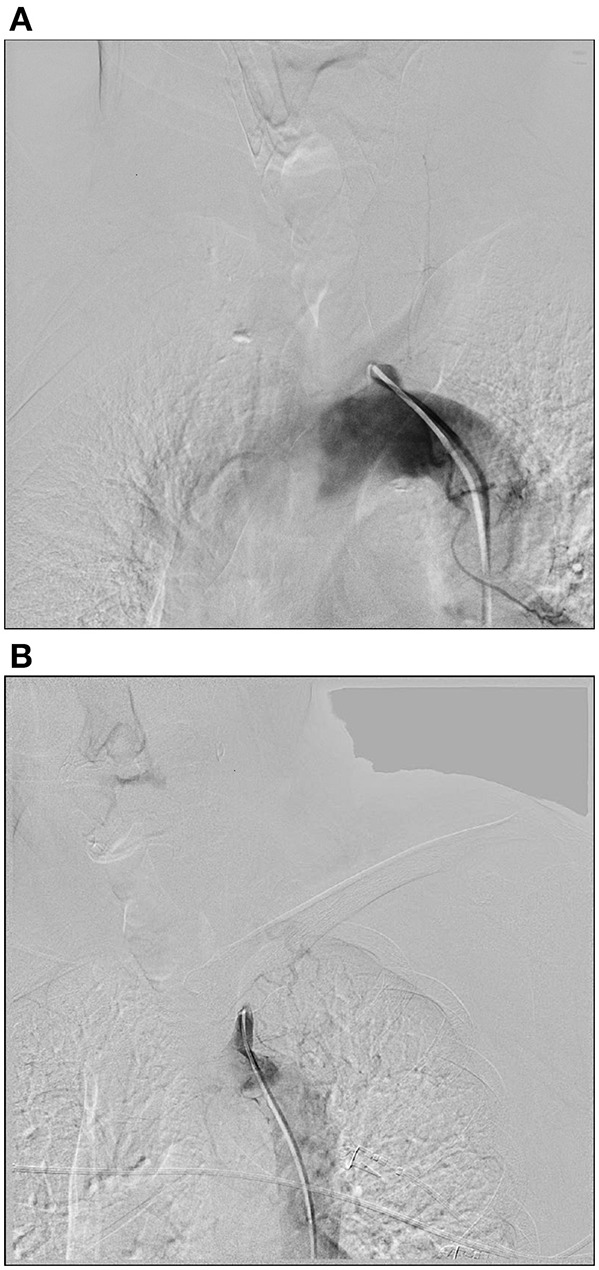
Two types of stumps. **(A)** The obtuse lesion means the stump were short and flat. **(B)** The beaklike lesion had a tip on the top of the stump.

We divided the stump into two tapes: obtuse and beaklike lesions. The obtuse lesion means the stump were short and flat. And the beaklike lesion had a tip on the top of the stump (Figure 1). Technical success was defined as <30% residual stenosis evaluated by post-treatment angiography. Clinical success was defined as resolution of symptoms and signs. Primary patency was defined as uninterrupted patency of the treated subclavian artery without any endovascular or open surgeries to maintain or restore patency. Restenosis was defined as ≥50% diameter reduction after primary treatment. Secondary patency was defined as the patency of a treated artery with secondary endovascular intervention or open surgery to restore the patency after restenosis happened.

### Statistical Analysis

Statistical analysis was performed with SPSS version 19.0. Kaplan–Meier analysis was used to evaluate the rates of primary patency and secondary patency.

## Results

### Technical Success and Procedural Details

In this study, the technical success rate was 91.3% (21/23). Procedural failures were due to unsuccessful guide wire passage through occlusive lesions via both antegrade and retrograde approaches in 2 patients. Clinical remission rate was 95.2% (20/21) after treatment. A patient who presented with vertigo felt no substantial improvement after treatment.

**Figure 2:**
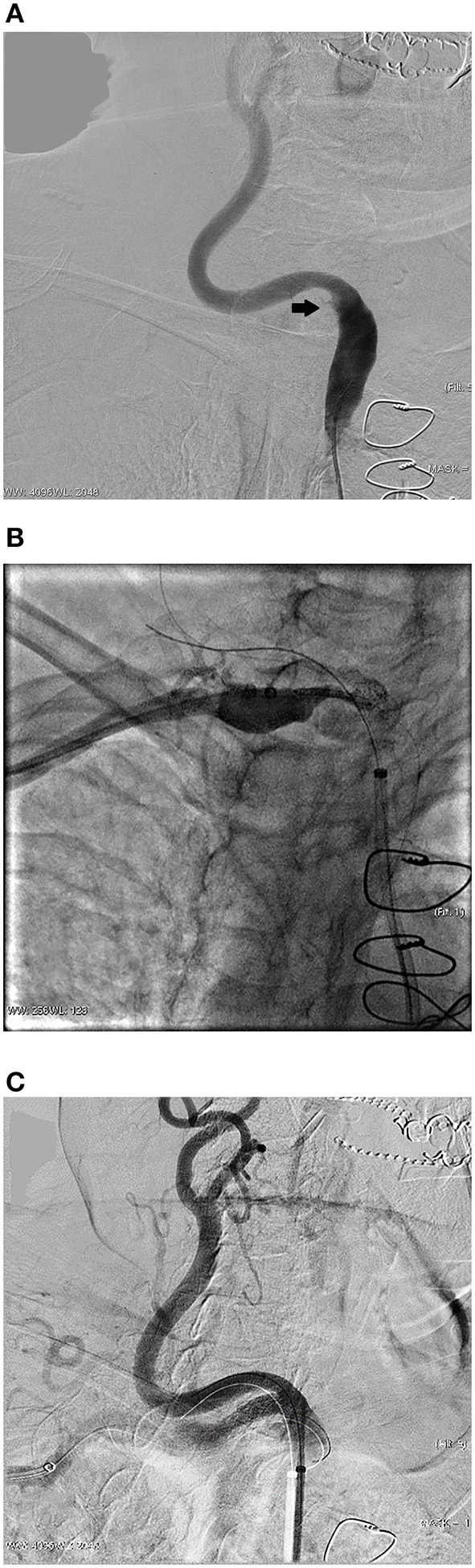
A representative case of successful retrograde recanalization. This was an 84 years old female patient who presented with vertigo for 1 year. **(A)** Angiography shows occlusion of the right subclavian artery ostium. **(B)** After failed antegrade recanalization, we attempted retrograde recanalization via the brachial artery. An emboli protection device was placed in the right internal carotid artery. Angiography was performed before deploying a balloon expandable stent to confirm the accurate location. **(C)** Angiography shows the perfect position of the stent, which completely covered the lesion but did not affect the blood flow of the right carotid artery.

The antegrade approach was chosen as the primary recanalization strategy in 22 cases, resulting in a success rate of 68.2% (15/22). In these 7 cases which we failed to pass-through by antegrade approach, we attempted the retrograde approach via the brachial artery on the affected side. And in 1 case, transfemoral access was not feasible because of bilateral iliac artery occlusion, so we attempted recanalization via the retrograde approach directly. This patient had arterial dissection of the left subclavian artery extended to the aortic arch. We would describe it in detail in the next section. The success rate of retrograde recanalization was 75.0% (6/8). For the both right side lesions, we used the embolic protection devices. Figure 2 shows a representative case of successful retrograde recanalization. During lesion treatment in the right subclavian artery ostium, we placed an embolic protection device in the right internal carotid artery to avoid embolism before we breakthrough the lesion.

For all these cases either antegrade or retrograde approach, we try to breakthrough the lesion with 0.035 inch super-slide hydrophilic guidewires (TERUMO) and 5F VER catheters (TERUMO) for supporting initially. For the failed cases, we choose the 0.018 inch Treasure 12 CTO guidewire (ASAHI) and CXI support catheter (COOK) as alternative. Eleven lesions were successfully recanalized with a 0.035 inch super-slide hydrophilic guidewire, while the other 10 lesions were crossed with a 0.018 inch CTO guidewire. The technical success rate for obtuse-stump and beaklike-stump group were 50.0% (6/12) and 90.0% (9/10), respectively.

We used guiding catheter (8F, Boston Scientific) in 5 cases and long sheath (7F, COOK) in other 18 cases to implant stents.

Table 3 lists details about the lesions, procedure approaches, guidewires, shapes of stump, balloons for pre-dilation and stent.

**Table 3:** Lesion and procedural details (mm).

**No**.	**Success**	**Side**	**Approach**	**Stump**	**1st GW**	**2nd GW**	**Balloon**	**Stent**
1	√	L	Ante → Retro	O	Super-slide		Synergy 8 ×40	Precise 9 ×30
2	√	L	Ante	O	Super-slide		Admiral 7 ×40	Precise 9 ×30
3	√	L	Ante	B	Super-slide	CTO	ClearStream 6 ×60	Smart 8 ×60
4	√	L	Retro	/	Super-slide	CTO	Admiral 6 ×60	Fluency 9 ×60
5	√	L	Ante	B	Super-slide		Admiral 7 ×40	Precise 9 ×30
6	√	L	Ante	B	Super-slide	CTO	Admiral 6 ×40	Precise 9 ×30
7	√	L	Ante	B	Super-slide	CTO	Bantam 6 ×60	GPS 8 ×60
8	√	L	Ante → Retro	B	Super-slide	CTO	Sterling 6 ×30	Precise 8 ×40
9	√	L	Ante	O	Super-slide		Admiral 6 ×80	Everflex 8 ×60
10	√	L	Ante → Retro	O	Super-slide	CTO	Sterling 5 ×30	Precise 8 ×40
11	√	L	Ante	O	Super-slide	CTO	Sterling 5 ×30	BLUE 7 ×18
12	√	R	Ante → Retro	O	Super-slide	CTO	Sterling 7 ×30	Precise 9 ×30
13	√	L	Ante	B	Super-slide		Admiral 6 ×60	GPS 8 ×80
14	√	L	Ante	O	Super-slide		FOXPLUS 6 ×40	Express LD 8 ×17
15	√	L	Ante	O	Super-slide		Admiral 6 ×40	GPS 8 ×60
16	√	R	Ante	O	Super-slide	CTO	Admiral 6 ×40	COMPLETE 8 ×60
17	√	L	Ante	B	Super-slide		Admiral 4 ×60	Scuba 8 ×30
18	√	L	Ante	B	Super-slide		Admiral 5 ×80	Dynamic 8 ×25
19	X	L	Ante → Retro	O	Super-slide	CTO		
20	X	L	Ante → Retro	O	Super-slide	CTO		
21	√	L	Ante	B	Super-slide		Admiral 7 ×40	Dynamic 8 ×25
22	√	L	Ante	B	Super-slide		Admiral 5 ×30	Precise 8 ×30
23	√	L	Ante → Retro	0	Super-slide	CTO	Admiral 6 ×40	Precise 9 ×30

*Stump: B for beaklike, O for obtuse*.

*Balloon*:

*Synergy (Boston Scientific), Admiral (Medtronic), ClearStream (ClearStream Technologies), Bantam (ClearStream Technologies), Sterling (Boston Scientific), FOXPLUS (Abbott)*.

*Stent*:

A. Self-expandable stent

*Precise (Cordis), Smart (Cordis), GPS (EV3), Everflex (EV3), COMPLETE (Medtronic)*.

B. Balloon expandable stent

*BLUE (Cordis), Express LD (Cordis), Scuba (INVATEC), Dynamic (Biotronik)*.

C. Cover stent

*Fluency (BARD)*.

### Adverse Events

**Figure 3:**
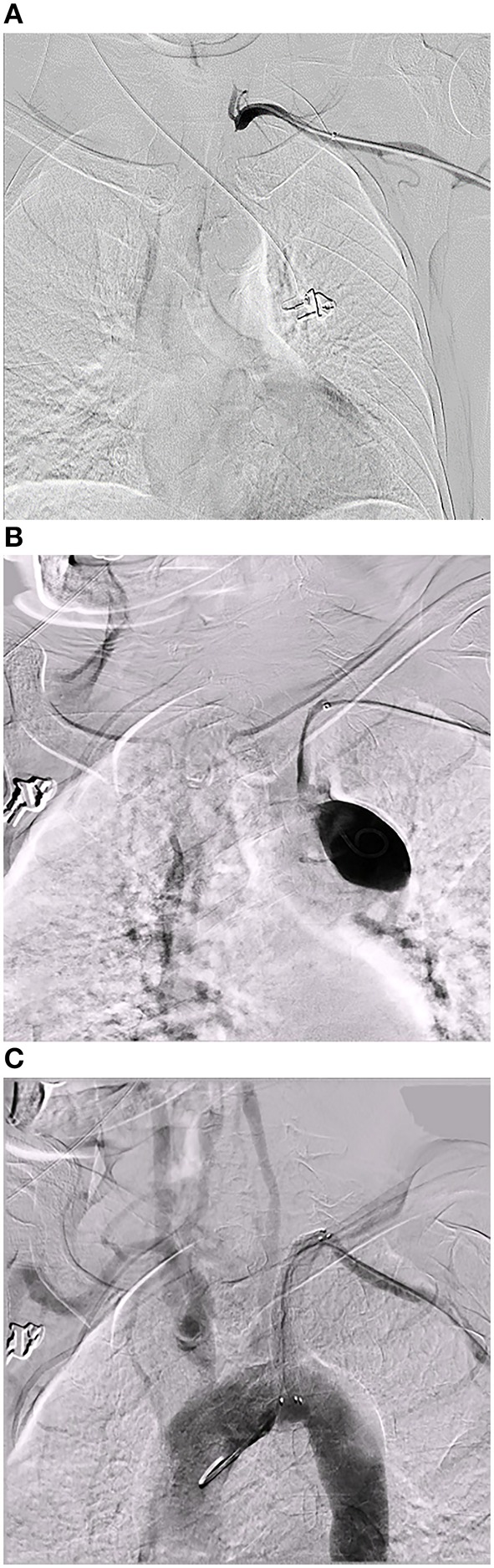
An arterial dissection. A 70 year-old male patient who presented with vertigo and left arm claudication for 2 years. CTA of the lower extremity showed bilateral iliac artery occlusion. **(A)** Angiography revealed a long segment occlusion at the beginning of the left subclavian artery. **(B)** After passing a guidewire through the lesion via the retrograde approach through the left brachial artery, angiography revealed an arterial dissection from the proximal segment of the left subclavian artery to the aortic arch. **(C)** A covered stent was implanted to treat the dissection, but the blood flow of the left vertebral artery was affected.

Neither perioperative death nor permanent neurological deficit was observed. In one case, transfemoral access was not feasible because of bilateral iliac artery occlusion, so we attempted recanalization via the retrograde approach through the left brachial artery. After the wire passed through the lesion, the patient complained of a tearing pain in the chest. Angiography revealed an arterial dissection from the proximal segment of the left subclavian artery to the aortic arch. The pain was relieved after implanting a covered stent. However, the origin of the left vertebral artery was also covered (Figure 3).

### Follow-Up

**Figure 4:**
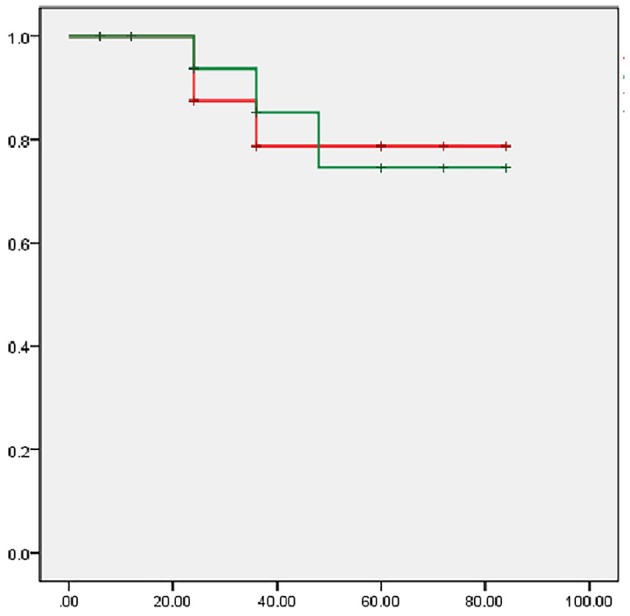
Kaplan–Meier analysis of cumulative primary and secondary patency rates. X-axis: follow-up time (month); Y-axis: patency rates; Red line: cumulative primary patency rate; Green line: cumulative second patency rate.

The follow-up time was 6 to 84 months (median, 36 months). Restenosis was detected in 3 cases. Among these cases, 2 cases with in-stent restenosis were found by ultrasound scanning at 24 month follow-up. Both patients developed vertigo again and then received percutaneous balloon angioplasty. The retreatment success rate was 100%. For the other patient, who had only mild upper extremity weakness, restenosis was detected at 36 month follow-up, but the patient declined endovascular treatment. Rates of estimate cumulative primary and secondary patency at 5 years were 74.6 and 78.8% by Kaplan–Meier analysis, respectively (Figure 4).

## Discussion

Patients with hemodynamically significant stenosis of the subclavian artery may present as subclavian steal syndrome, with the symptoms of upper limb ischemia, or, rarely, as coronary steal syndrome. In this study, >50% of the study patients had symptoms of vertebrobasilar insufficiency, while 20% had symptoms of upper extremity ischemia. This pattern differs from other study which reported arm claudication is the most common complaint (7).

It has been reported that the technical success rate of endovascular therapy for subclavian artery stenosis is very high. Song et al. reported that the technical success rate was 97.3% in 148 patients with balloon-expandable stents (8). However, the technical success rate of endovascular treatment for CTO lesions in the subclavian artery was lower. Babic et al. reported that the successful recanalization rate was 82.1% (9). The success rate in this study was higher than that reported by Babic et al. The key goal of this study was to determine the optimal method to improve the rate of successful recanalization. During the procedure, we placed a guiding catheter or a long sheath at the stump of the occluded subclavian artery to provide stronger support force. Artery dissection is one of the serious complications in the procedures of CTO lesions. According to the location of the dissection, the risk of local subclavian artery dissection is less than that propagate into the aorta. Anatomically, antegrade approach is not easy to cause aortic dissection. So antegrade approach is the preferred approach to tackle subclavian artery CTO. We used the retrograde approach as an alternative, which has been used when the antegrade approach fails (10). In most cases, in which antegrade recanalization failed, there was successful revascularization via the retrograde approach in this study. After recanalization, angiography was required to confirm that the guidewire was in the true lumen to ensure dissection did not happen. The 0.035 inch super-slide hydrophilic guidewire is our workhorse to recanalize CTO lesions. When the shape of stump was obtuse, super-slide hydrophilic guidewire might not get enough support force to drill through the lesion. CTO wires are rather different from super-slide hydrophilic guidewires. This type of wire is capable of drilling the fibrous caps of lesions, leading to an increased rate of successful recanalization. Thus CTO wires pass through occlusive lesions with greater ease, especially in cases of occlusion without obvious stump. We divided lesions for the stump shapes into two groups. The technical success rate of beaklike-stump group was higher than obtuse group. The tips of beaklike-stump lesions were always the former lumen. We could “find” the right way to distal true lumen with guidewire easily. We could also acquire more support force by the cooperation of guidewire and catheter. The previous literature has not stated this difference (9, 10).

Left subclavian artery stenosis is more prevalent than right subclavian artery stenosis, with the left to right ratio being between 2:1 and 4:1 (11, 12). The majority of lesions in this study were in the left subclavian artery. Because of the unique anatomical features, endovascular treatment of right subclavian artery stenosis always has additional difficulties, including stent implanting, stent location, and risk of right-hemisphere cerebral infarction. Implantation of a balloon-expandable stent under angiography could solve the problem of stent location. Balloon-expandable stents can be accurately located at the ostium of the right subclavian artery. Embolic protection devices, which are placed in the distal segment of the right internal carotid artery before dilation, could reduce infarction. In most cases of left subclavian artery stenosis, embolization of the carotid artery is not a common problem. Both self-expandable stents and balloon-expandable stents are feasible.

The long-term patency rate in our study was not different from the previously reported patency rate of atherosclerotic subclavian artery stenosis (13). Restenosis is a common adverse event of stent implantation. If the patient had recurrent symptoms, it should be considered to receive revascularization again. Endovascular recanalization can achieve an acceptable long-term secondary patency.

## Limitations

The retrospective nature of this study is a primary limitation. Some follow-up results were based on ultrasound examination and there was operator bias. In addition, all cases were enrolled from a single-center. Moreover, a small sample size might influence the accuracy of long-term patency analysis.

## Conclusion

Taken together, endovascular treatment of CTO of the subclavian artery has a high technical success rate and an acceptable long-term patency rate. Recanalization is the key step of the procedure. The antegrade approach should be the first choice, while the retrograde approach, with a potential risk of causing aortic dissection, could be considered as an alternative. Endovascular treatment is a safe and effective treatment for subclavian artery chronic total occlusion.

## Data Availability Statement

The datasets generated for this study are available on request to the corresponding author.

## Author Contributions

GN: responsible for writing this manuscript, diagnosis and procedures of patients. ZY and BZ: data collection and data analysis and interpretation. MY: conception or design of the work, critical revision of the article, and final approval of the version to be published.

## Conflict of Interest

The authors declare that the research was conducted in the absence of any commercial or financial relationships that could be construed as a potential conflict of interest.
